# CD4+ conventional T cells-related genes signature is a prognostic indicator for ovarian cancer

**DOI:** 10.3389/fimmu.2023.1151109

**Published:** 2023-03-31

**Authors:** Tian Hua, Deng-xiang Liu, Xiao-chong Zhang, Shao-teng Li, Peng Yan, Qun Zhao, Shu-bo Chen

**Affiliations:** ^1^ Department of Gynecology, Affiliated Xingtai People Hospital of Hebei Medical University, Xingtai, China; ^2^ Department of Oncology, Affiliated Xingtai People Hospital of Hebei Medical University, Xingtai, China; ^3^ Department of Oncology, The Second Affiliated Hospital Of Xingtai Medical College, Xingtai, China; ^4^ Department of Oncology, Hebei Medical University, Fourth Hospital, Shijiazhuang, China; ^5^ Hebei Key Laboratory of Precision Diagnosis and Comprehensive Treatment of Gastric Cancer, Shijiazhuang, China

**Keywords:** ovarian cancer, CD4+ conventional T cells, prognostic signature, tumor microenvironment, immunotherapy

## Abstract

**Introduction:**

It is believed that ovarian cancer (OC) is the most deadly form of gynecological cancer despite its infrequent occurrence, which makes it one of the most salient public health concerns. Clinical and preclinical studies have revealed that intratumoral CD4+ T cells possess cytotoxic capabilities and were capable of directly killing cancer cells. This study aimed to identify the CD4+ conventional T cells-related genes (CD4TGs) with respect to the prognosis in OC.

**Methods:**

We obtained the transcriptome and clinical data from the Cancer Genome Atlas (TCGA) and Gene Expression Omnibus (GEO) databases. CD4TGs were first identified from single-cell datasets, then univariate Cox regression was used to screen prognosis-related genes, LASSO was conducted to remove genes with coefficient zero, and multivariate Cox regression was used to calculate riskscore and to construct the CD4TGs risk signature. Kaplan-Meier analysis, univariate Cox regression, multivariate Cox regression, time-dependent receiver operating characteristics (ROC), decision curve analysis (DCA), nomogram, and calibration were made to verify and evaluate the risk signature. Gene set enrichment analyses (GSEA) in risk groups were conducted to explore the tightly correlated pathways with the risk group. The role of riskscore has been further explored in the tumor microenvironment (TME), immunotherapy, and chemotherapy. A risk signature with 11 CD4TGs in OC was finally established in the TCGA database and furtherly validated in several GEO cohorts.

**Results:**

High riskscore was significantly associated with a poorer prognosis and proven to be an independent prognostic biomarker by multivariate Cox regression. The 1-, 3-, and 5-year ROC values, DCA curve, nomogram, and calibration results confirmed the excellent prediction power of this model. Compared with the reported risk models, our model showed better performance. The patients were grouped into high-risk and low-risk subgroups according to the riskscore by the median value. The low-risk group patients tended to exhibit a higher immune infiltration, immune-related gene expression and were more sensitive to immunotherapy and chemotherapy.

**Discussion:**

Collectively, our findings of the prognostic value of CD4TGs in prognosis and immune response, provided valuable insights into the molecular mechanisms and clinical management of OC.

## Introduction

Among all gynecological malignancies, ovarian cancer causes the most deaths, and it is estimated that ovarian cancer accounts for 5% of all cancer deaths in women. In 2023, There will be 19,710 new cases and 13,270 new deaths because of OC in the United States (1). The reason for death was mainly due to late-stage diagnosis (2). Given the genetic and non-genetic risk factors of OC, OC was considered a particularly challenging cancer to overcome. Over the past few decades, a higher degree of radicality has been implemented in ovarian cancer surgery (3). In addition, homologous recombination repair deficiency (HRD) and BRAC1/2 gene mutations testing also optimize PARP inhibitor (PARPi) use aimed to improve the benefit of patients even in the most advanced stages of the disease (4, 5). Although the treatments have reduced OC-related deaths to a certain extent, patient outcomes remained unfavourable. Therefore, it was necessary to develop new prognostic signatures and molecular biomarkers.

As a result of comprehensive sequencing efforts over the past decade, we have learned about the genomic landscape of common forms of human cancer. Many studies have focused on the promotion or inhibition of cancer genes. High throughput screening, such as RNAi and CRISPR, were used to identify cancer dependency genes and their relationships to genetics, expression, regulatory mechanism, and therapeutic potential (6, 7). New immunotherapeutics have been developed due to advances in cancer immunology (8, 9). Cytotoxic T cells were essential effectors of anti-tumor immunity (9). Zheng et al. demonstrated the tumor infiltrating T cell compendium, dynamics, and regulation in many cancer types by single-cell RNA-seq (scRNA-seq). They compared the phenotype and tissue distribution of CD8+ T cell and CD4+ T cell among blood, normal tissue, tumor tissue. CD8+ T cell has 17 different subclusters, such as ISG+CD8+ T cell and tissue-resident memory T cells (T_rm_). CD4+ T cell has 24 different subclusters, such as IL26+Th17 and TNFRSF9+Treg. Terminally differentiated effector memory (T_emra_) and naïve T cells (T_n_) were enriched in blood between CD8+ T cell and CD4+ T cell. Most tested cancer types exhibited a notable degree of motility for both CD8+ and CD4+ T_emra_ cells between blood and normal or tumor tissues. The classical CD4+ T cell marker were CD3D, CD3E, CXCR4, IL7R, LTB, TRBC2 (10). While tumor killing was considered to be CD8+ T cell function, the majority of previous understanding of the functionality of CD4+ T cells came from studies about anti-viral immunity (11, 12). CD4+ T cells recognized cognate viral antigens in a major histocompatibility complex class II (MHC class II) -restricted manner (13). Within the cancer context, multiple lines of evidence pointed to an important role for CD4+ T cells in immune responses to cancer immunotherapy (14–19). For example, Martens et al. indicated that increased CD4+ T cell percentages at 8-14 weeks positively correlated with the expected pharmacodynamic effect (14). There was also more direct evidence of the therapeutic benefits of CD4+ T cells in neoantigen vaccination, with CD4+ T cell responsed to neoantigen vaccines being more prevalent than CD8+ T cell responses (20, 21). CD4+ T cells have also played a pivotal role in cancer induced by viruses. The expression of the EBV signaling protein LMP1 in B lymphocytes triggered CD4+ T cell responses against various tumor-associated antigens (22). Thanks to the rapid development of single-cell sequencing experiments and analytical techniques, some studies found that CD4+ conventional T cells-related lncRNAs signature was associated with hepatocellular carcinoma, breast cancer prognosis, therapy, and tumor microenvironment (23, 24). However, few studies have focused on the prognosis of CD4+ conventional T cells-related genes in OC.

As a result of bulk sequencing, we averaged the genetic and expression profiles of the different tumor subpopulations (25). New technologies based on single-cell sequencing have opened new avenues for understanding intra-tumoral heterogeneity and capturing different tumor states with unprecedented resolution and scale (26, 27). In the present study, based on bulk and single-cell sequencing datasets, we established a prognostic signature based on CD4TGs for OC. Clinical features, overall survival (OS), progress-free survival (PFS), tumor microenvironment, immunotherapy, and chemotherapy were evaluated between high and low riskscore subpopulations.

## Materials and methods

### Data acquire

We downloaded RNA-seq gene expression data of transcripts per million (TPM) values, clinical information, and masked annotated somatic mutation datasets of OC (tumor type was high-grade serous ovarian cancer) from The Cancer Genome Atlas (TCGA, https://portal.gdc.cancer.gov/). Only primary solid tumor patients were kept in the analysis. Single-cell RNA-seq data (GSE118828, GSE147082) and prognosis validation datasets (GSE26193, GSE63885, GSE140082) were obtained from GEO databases (https://www.ncbi.nlm.nih.gov/geo/) (28–34). TCGA data tpm value was log2(x+1) transformed and z-scored, GEO matrix was z-scored.

### Identifying CD4Tconv-related differential expressed genes OC

The Tumor Immune Single Cell Hub 2 (TISCH2) was a resource of single-cell RNA-seq (scRNA-seq) data from human and mouse tumors, which conducted comprehensive characterization of gene expression in the TME (35). We firstly obtained CD4TGs from TISCH2 with the criteria (|log2FC| > 1 and Adjusted p-value < 0.05). We then intersected the genes in two scRNA-seq GEO datasets, the TCGA dataset, and three external validation GEO datasets. 265 CD4TGs were harvested in the final.

### Comprehensive analysis of single-cell datasets and cell cluster annotation

scRNA-seq dataset analysis was performed using the R package Seurat (v4.1.1) (36). UMAP analysis was done through Seurat’s built-in function RunUMAP and umap-learn’s built-in algorithm, and the Leiden algorithm. Finally, dimplot, featureplot, violin, and dotplot were used for visualization. The metabolic scores of different clusters of cell subtypes were calculated by the R package scMetabolism with the method AUCell in reactome pathway (37). The results of the scMetabolism calculations were integrated and visualised with dotplot pheatmap to demonstrate the metabolism of different clusters of cell subtypes. We also used AddModuleScore function to calculate the risk score in cell subsets level and sample level of the two single-cell GEO datasets.

### Construction of CD4+Tconv-related genes riskscore signature

To screen genes associated with OS in OC patients, univariate Cox regression, least absolute shrinkage and selection operator (LASSO) regression, and multivariate Cox regression were executed sequentially to figure out eleven meaningful CD4+Tconv-related genes. Based on their expression and corresponding multivariate Cox regression coefficients, the riskscore was calculated as follows:

Riskscore = ∑multivariate Cox regression coefficient (gene i) **x** gene expression value (gene i). The patients were divided into high-risk and low-risk subgroups by median riskscore in TCGA datasets. We also randomly splited the TCGA dataset into train and test datasets at a 1:1 ratio to predict OS by Kaplan-Meier (K-M) survival analysis. The patients were divided into high-risk and low-risk subgroups by best cutoff riskscore value (R package “survminer”) to validate OS or PFS by Kaplan-Meier (K-M) survival analysis in validation GEO datasets.

### Nomogram and calibration

In the whole TCGA dataset, time-dependent receiver operating characteristic (ROC) curve analysis was conducted to determine the prognostic value of riskscore over time. We also explored the role of the riskscore in different clinical subgroups (age, grade, stage, tumor residual size). The nomogram was constructed using multivariate Cox regression analysis by integrating clinical information and riskscore (R package “regplot”), and calibration curves were used to check the accuracy of the nomogram. The clinical benefits conferred by prognostic evaluation of the nomogram were further compared using decision curve analysis (DCA).

### Functional enrichment analysis

Tool GSEA v4.3.2 from the MSigDB database (http://software.broadinstitute.org/gsea/msigdb/) was used to find the highly related GO and HALLMARK pathways between high-risk and low-risk subgroups based on the criterion of selection (FDR q-value < 0.25, Nominal p-value < 0.05 and |NES| >= 1.5) (38, 39).

### Tumor microenvironment and immune infiltration level analysis

The “estimate” package was used to determine immune scores, stroma scores, and estimate scores. The abundance of immune cells was estimated using TIMER (40). Immunophenoscore (IPS) derived from The Cancer Group Atlas(TCIA, https://tcia.at/home) was used to predict the response to checkpoint blockade (41, 42). A single-sample gene set enrichment analysis (ssGSEA) was performed to quantify immune cells and immune function (R packages: “GSVA” and “GSEABase”). Immune subtypes information was derived from the previous study (43).

### Drug sensitivity analysis

The origin data of chemotherapy response was from Genomics of Drug Sensitivity in Cancer (GDSC version 2) (https://www.cancerrxgene.org/) (44), and we downlaoded curated data from https://osf.io/temyk. R package oncoPredict was used to predict the chemotherapy response difference between high-risk and low-risk subgroups (45).

### Quantitative real-time PCR

RNA was extracted from ISOE, SKOV3, and A2780 cellines using the Trizol and then reverse-transcripted into cDNA. Primers were designed and obtained from the genewiz company. For real-time PCR, cDNA was used as template, and the PCR reaction was performed using QuantStudio(TM) 7 Flex System. The primer sequences used were listed in **Supplementary Table 9**.

### Statistical analysis

All statistical analyses were performed by R software 4.2.2 or GraphPad 8. p-value < 0.05 was deemed to be statistically significant unless noted otherwise. Ns, *, **, ***, and **** stand for p-value >0.05, p-value <=0.05, pvalue <=0.01, pvalue <=0.001, and pvalue <=0.0001, separately. Survival analysis was carried out using the R packages “survival” and “survminer”. We used the Wilcoxon test when comparing two groups and Kruskal-Wallis when comparing more than two groups.

## Results

**Figure 1:**
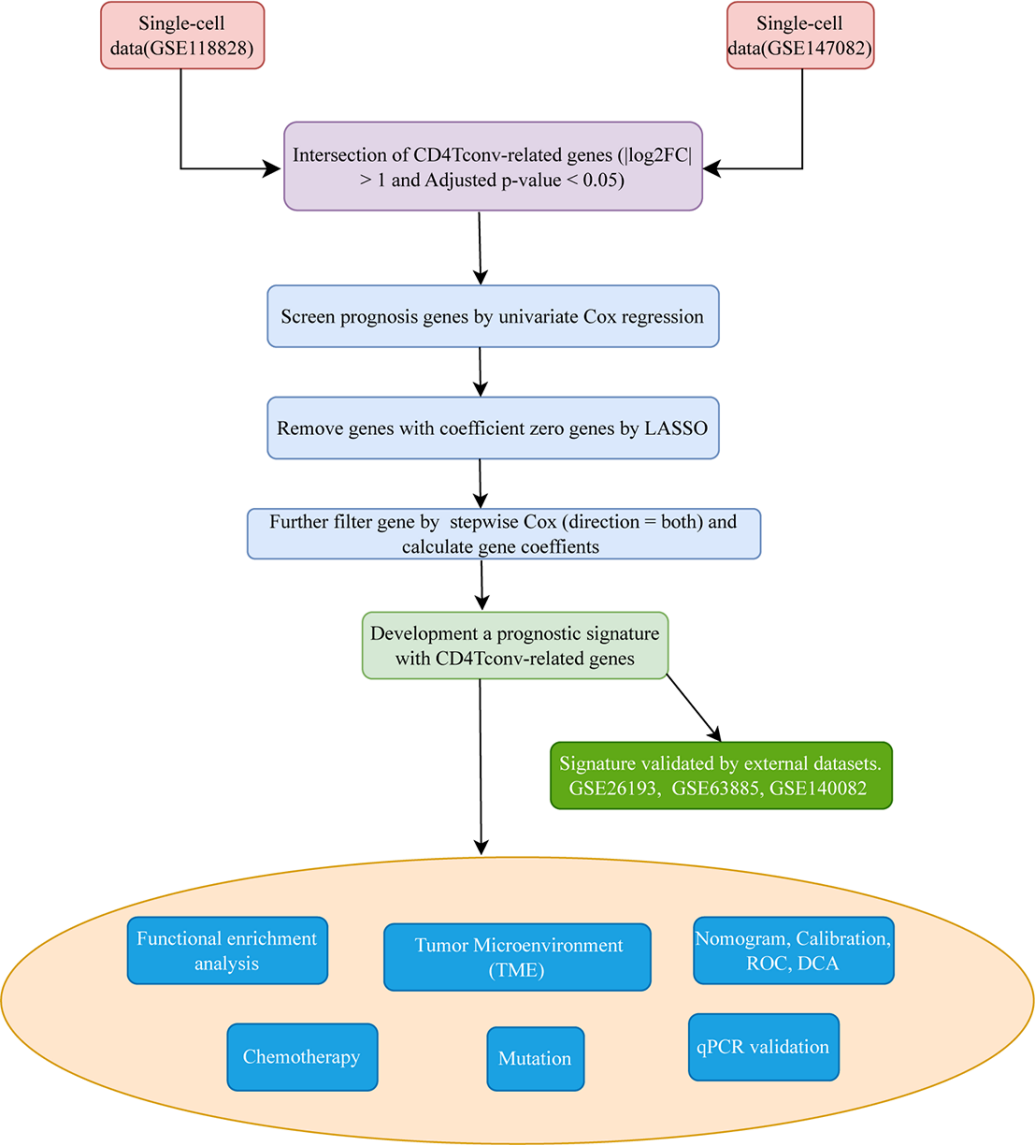
Workflow diagram. The specific workflow graph of data analysis of the study.

The whole workflow of this study was shown in **Figure 1**. We firstly obtained two single-cell sequencing (scRNA-seq) datasets from online database, and intersected the significant differential expression genes. Then TCGA bulk-seq data was used to screen prognosis genes by univariate Cox regression. LASSO algorithm was conducted to remove genes with coeffient zero. We furtherly filter gene by stepwise Cox (direction = both), calculated gene coeffients and finally built the risk model. We also made more tumor prognosis-related analyses.

### Analysis of OC single-cell sequencing data

**Figure 2:**
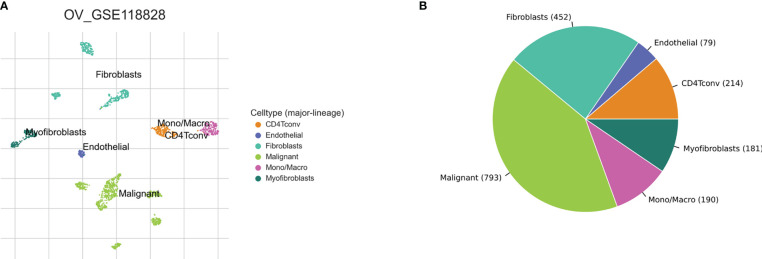
Ovary cancer single-cell data analysis based on the GSE118828 dataset. **(A)** The UMAP plots with cells coloured by cell type were displayed. **(B)** The pie plot showed the cell number distribution of each cell type.

**Figure 3:**
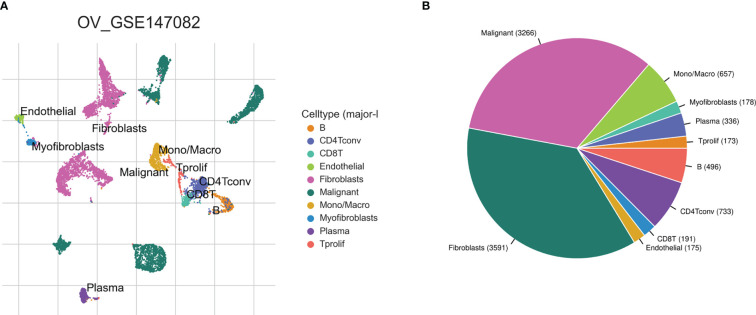
Ovary cancer single-cell data analysis based on the GSE147082 dataset. **(A)** The UMAP plots with cells coloured by cell type were displayed. **(B)** The pie plot showed the cell number distribution of each cell type.

Based on the TISCH2 database, we obtained two scRNA-seq datasets, GSE118828 (SMART-seq2 platform) and GSE147082 (Drop-seq platform) and re-analysed using R package Seurat. The markers for each cell type were listed in **Supplementary Table 1** and shown in **Supplementary Figure S1**. It was easy to find the classical marker, CD3D, CD3E, CXCR4, IL7R mainly expressed on CD4Tconv (CD4+ conventional T) subset (**Supplementary Figure 1**). As shown in **Figures 2A, B**, **3A, B**, we could find that CD4Tconv ranked third proportion in two datasets, just behind fibroblasts and malignant cells. In dataset GSE118828, the GSEA analysis of KEGG pathways showed CD4Tconv was significantly enriched in nature killer cell mediated cytotoxicity, T cell receptor signaling pathway, JAK-STAT signaling pathway, complement and coagulation cascades pathways (**Supplementary Figures 2A, B**). In dataset GSE147082, the GSEA analysis of KEGG pathways showed CD4Tconv was significantly enriched in nature killer cell mediated cytotoxicity, JAK-STAT signaling pathway, T cell receptor signaling pathway, ecm receptor inter pathways (**Supplementary Figures 2C, D**). These results suggested that CD4Tconv played a vital role in OC immunity-related pathways and was worthy of further study. We also investigated the metabolic status of different clusters of cell types. The result showed that CD4Tconv were enriched in metabolism of RNA, metabolism of amino acids and derivatives, selenoamino acid metabolism, phospholipid metabolism, pi metabolism, inositol phosphate metabolism pathways in dataset GSE118828 (**Supplementary Figure 3A**). This same metabolism result was also validated in dataset GSE147082 (**Supplementary Figure 3B**).

### Development and validation of prognostic signatures associated with CD4+TGs in OC

**Figure 4:**
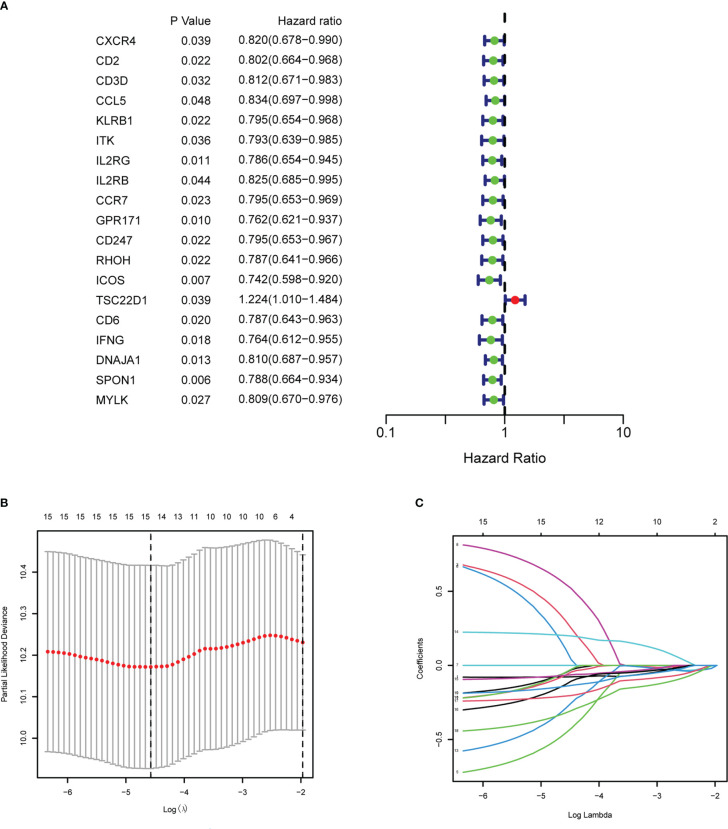
Establishment of the CD4+ conventional T cells-related genes signature in ovary cancer. **(A)** Prognosis-associated genes were extracted by univariate Cox regression analysis. **(B)** Ten-fold cross-validation for variable selection in LASSO regression analysis. **(C)** LASSO coefficient profile of candidate genes.

**Figure 5:**
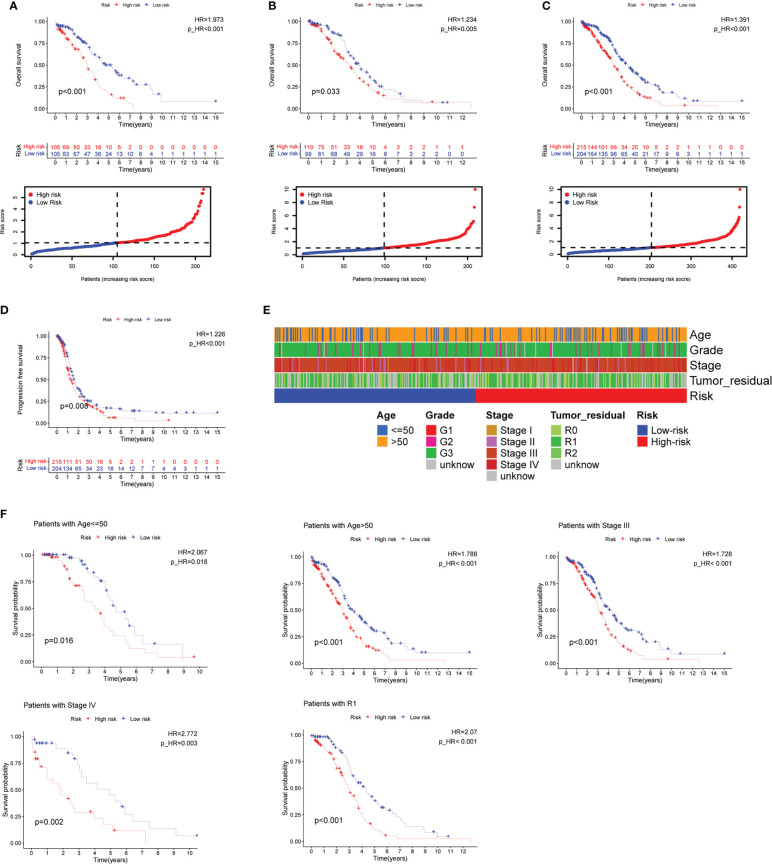
Prognosis value of the eleven CD4+ conventional T cells-related genes signature in the training, testing, and whole TCGA datasets. **(A–C)** Overall survival (OS) analysis in the training, testing, and whole TCGA datasets. **(D)** Progress-free survival (PFS) in the whole TCGA dataset. **(E)** Clinical information comparison between the high-risk and low-risk groups. **(F)** The prognostic value was stratified by the age, stage, and tumor residual size between high-risk and low-risk subgroups in the whole TCGA dataset.

**Figure 6:**
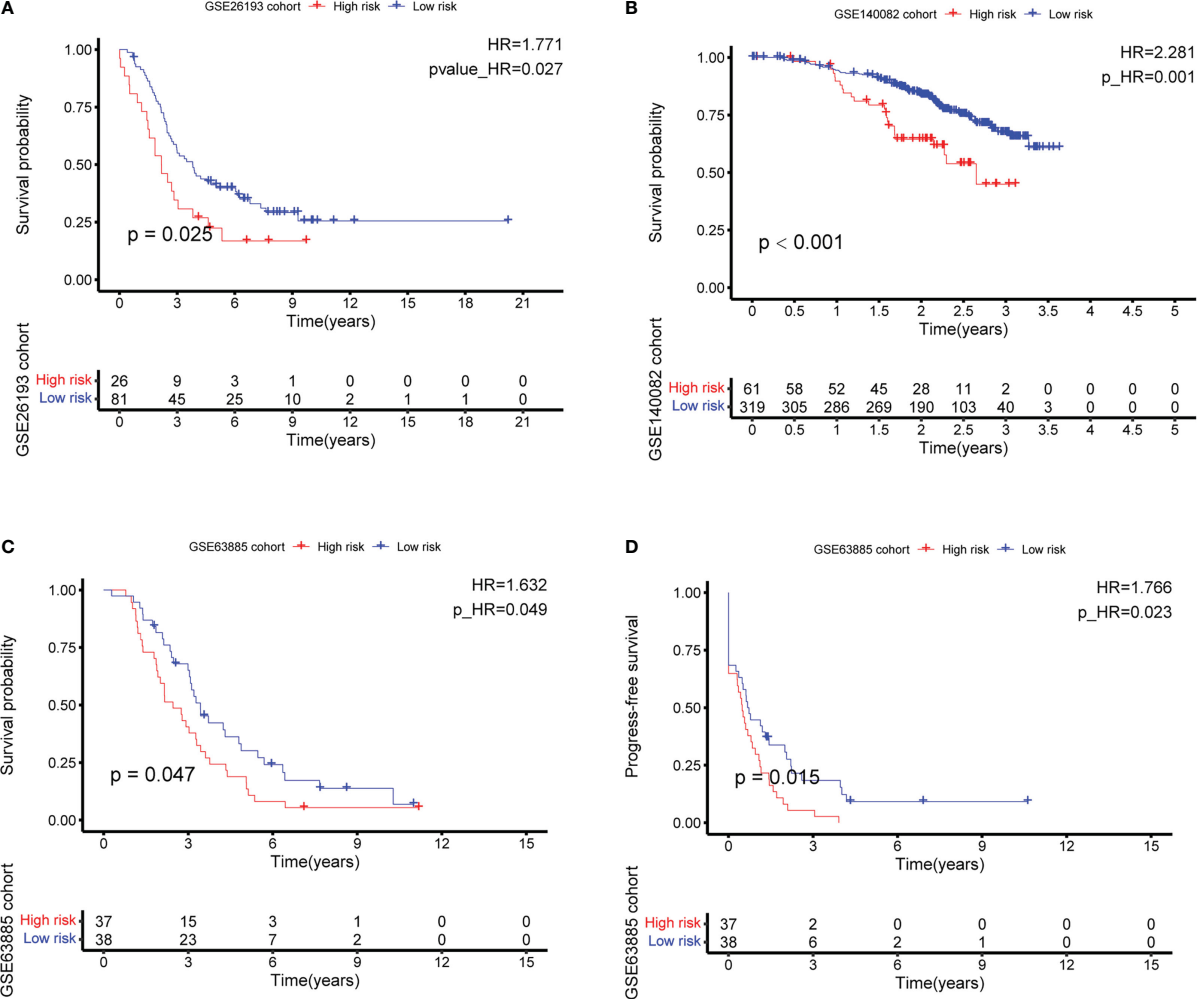
External validation of the CD4+ conventional T cells-related genes signature by best cutoff riskscore value. **(A–C)** Overall survival (OS) analysis in GSE26193, GSE140082, and GSE63885. **(D)** Progress-free survival (PFS) analysis in GSE63885.

**Figure 7:**
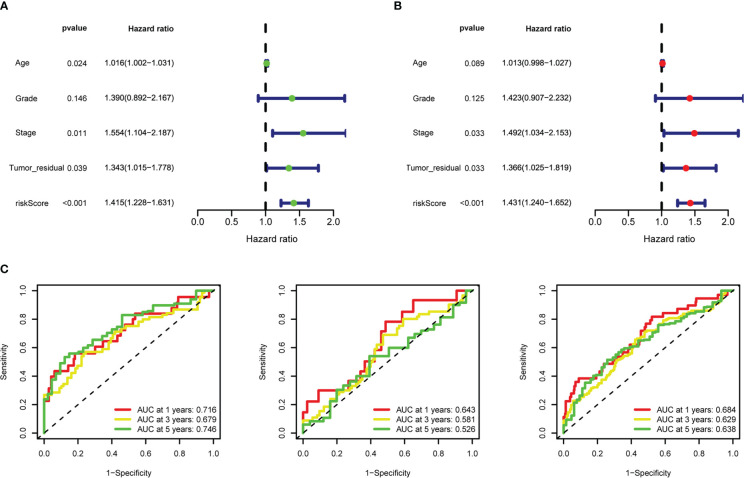
Riskscore as an independent prognostic factor. **(A)** Univariate Cox regression analysis of riskscore, age, stage, grade, tumor residual size. **(B)** Multivariate Cox regression analysis of riskscore, age, stage, grade, tumor residual size. **(C)** 1-, 3-, and 5-year time-dependent receiver operating characteristic (ROC) curves of the training, testing, and whole datasets, respectively.

After intersecting the genes in two scRNA-seq GEO datasets, the TCGA dataset, and three external validation GEO datasets. 265 CD4TGs were harvested finally. The genes list was in **Supplementary Table 2**. We first used univariate Cox regression analysis to screen significant genes in OS and found nineteen genes. The list of the genes was in **Supplementary Table 3**, and the forest plot was shown in **Figure 4A**. To narrow the list of the genes and get a more robust model, we furtherly conducted the LASSO algorithm according to the optimum lambda value and multivariate Cox regression analyses (**Figures 4B, C**). eleven genes were selected and generated the riskscore model in the final. The riskscore was calculated as follows: riskscore = (0.678 * CD3D expression) + (-0.897 * KLRB1 expression) + (0.535 * ITK expression) + (0.827 * IL2RB expression) + (-0.261 * CCR7 expression) + (-0.633 * ICOS expression) + (-0.619 * TSC22D1 expression) + (-0.413 * IFNG expression) + (-0.298 * DNAJA1 expression) + (-0.464 * SPON1 expression) + (-0.195 * MYLK expression). We splitted the internal validation TCGA dataset into train and test datasets at a ratio of 1:1. According to the median riskscore, OC patients were divided into high-risk and low-risk subgroups in the TCGA dataset. The results indicated that the high-risk group had a poorer prognosis in the train, test, and whole datasets (**Figures 5A–C**). In addition, we found the PFS also was significant between high-risk and low-risk subgroups in the TCGA whole dataset (**Figure 5D**). To avoid the difference of prognosis caused by the difference in clinical data, we compared the clinical features (age, grade, stage, tumor residual size) between high-risk and low-risk subgroups in the TCGA whole dataset and found there was no significant difference (**Figure 5E**), the statistic comparison result was in **Supplementary Table 4**. The detailed clinical information was in **Supplementary Table 5**. Thus proving the difference in prognosis was due to our risk signature instead of the imbalance in clinical data grouping. Additionally, we evaluated riskscore in different clinical characteristics to further develop the application. Age, stage III, stage IV, and R1 were significant prognostic between high-risk and low-risk subgroups in the TCGA whole dataset (**Figure 5F**). The above analyses were mostly based on only the TCGA dataset. We seeked some external datasets to validate the model to test the accuracy and robustness of the model. It could be seen that the OS were all significant in three independent GEO datasets based on the best cutoff in the riskscore, GSE26193 (p = 0.025), GSE140082 (p < 0.001), GSE63885 (p = 0.047) (**Figures 6A–C**). We also found that the high-risk group has a poorer PFS, consistent with the TCGA whole dataset (**Figure 6D**). To test whether A can be an independent prognostic factor, we combined clinical features (age, grade, stage, tumor residual size) and our pre-calculates riskscore into an integrated analysis. The univariate Cox regression analysis result showed the riskscore was significant (p < 0.001), and the hazard ratio was 1.415 (95% confidence interval, 1.228-1.631) (**Figure 7A**). The multivariate Cox regression analysis result showed the riskscore was an independent significant prognosis factor (p < 0.001), and the hazard ratio was 1.431 (95% confidence interval, 1.240-1.652) (**Figure 7B**). Time-dependent ROC analysis was performed to evaluate the predictive ability of the risk signature. The area under the curve (AUC) values at 1, 3, and 5 years for predicting OS were 0.716, 0.679, 0.746 in the TCGA train dataset, 0.643, 0.581, 0.526 in the TCGA test dataset, 0.684, 0.629, 0.638 in the TCGA whole dataset respectively (**Figure 7C**). ROC curves were also compared with other previous established risk models including a panel of three lncRNAs signature (AC136601.1

LINC02273 AC011445.1) (46) (**Supplementary Figure 4A**), a panel of five lncRNAs signature (GAS5, HCP5, PART1, SNHG11, SNHG5) (47) (**Supplementary Figure 4B**), a panel of six lncRNAs signature (AC006001.2, LINC02585, AL136162.1, AC005041.3, AL023583.1, LINC02881) (48) (**Supplementary Figure 4C**), a panel of eight mRNAs signature (JAK2, IL2RG, EEF1E1, UBB, EPS8, FOXO1, STAT5A, PAPPA) (49) (**Supplementary Figure 4D**), a panel of twelve mRNAs signature (CLDN4, EPCAM, MCM3, CXCL13, MIF, FOXO1, UBB, SEC22B, TCEAL4, ECI2, OGN, CFI) (50) (**Supplementary Figure 4E**). By comparing

the area under the curve (AUC) of ROC in 1 year, 3 years and 5 years. The detailed risk genes expression, riskscore and risk group were in **Supplementary Table 6**. We found that the predictive performance of our signature exceeded all the above risk models.

### Analyzing and estimating nomogram and risk gene expression

To predict the survival risk of OC patients and improve the clinical utility of the risk model, we created a nomogram based on all OC patients with riskscore and four other critical clinical features of OC to calculate an integrated point for each patient in the TCGA cohort. The result demonstrated that the nomogram point could accurately quantify survival rates (**Supplementary Figure 5A**). The calibration curves showed that the actual OS rates at 1-, 3-, and 5-year of patients and those estimated by the nomogram were close (**Supplementary Figure 5B**). The decision curve analysis (DCA) result suggested that the net rate of return for the OS rates evaluated by the combined risk model performed better than the other clinical characteristics (**Supplementary Figure 5C**). We explored the expression levels of the genes selected for risk pattern analysis in two single-cell datasets GSE118828 and GSE147082 by dotplot and violin plots (**Supplementary Figures 6A–D**). Consistently, most of risk genes (such as CD3D, KLRB1, ITK, CCR7 and ICOS) were up-regulated in CD4Tconv, while other risk genes (such as TSC22D1, SPON1 and MYLK) were down-regulated in CD4Tconv. The risk score calculated by AddModuleScore function was displayed in cell subsets level and sample level, CD4Tconv cells had relatively high level of risk scores (**Supplementary Figures 6E, F**). Besides, the expression of risk genes was also analyzed in TCGA dataset (**Supplementary Figure 7A**), along with validation cohorts GSE63885 (**Supplementary Figure 7B**), GSE26193 (**Supplementary Figure 7C**) and GSE140082 (**Supplementary Figure 7D**). We also analyzed the risk gene expression in two ovarian cancer celllines (SKOV3, A2780) and one normal ovarian celline (ISOE), the results were in **Supplementary Figure 8A**.

### Functional enrichment analysis of the 11 CD4TGs risk model

**Figure 8:**
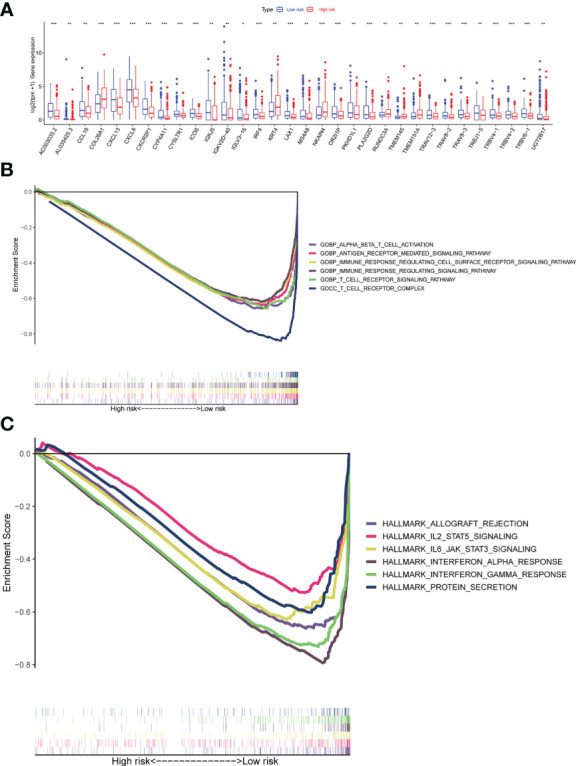
Gene set enrichment analyses (GSEA) of the Risk Groups. **(A)** Differential gene expression levels between high-risk and low-risk groups. **(B)** Highly enriched GO terms in the low-risk group. **(C)** Highly enriched Hallmark pathways in the low-risk group (all p < 0.05; FDR <0.25; |NES| > 1.5). ns, p > 0.05; *p <= 0.05; **p <= 0.01; ***p <= 0.001; ****p <= 0.0001.

To examine differences in biological function between high-risk and low-risk groups based on the riskscore. We first screened the differential genes among high-risk and low-risk groups with the following criteria: |logFC| ≥ 0.5 and a false discovery rate (FDR) < 0.05. The differential gene expression comparison was shown in **Figure 8A**. The detailed differential genes information was in **Supplementary Table 7**. GSEA software was used to search for GO and HALLMARK terms across the whole TCGA dataset in high-risk and low-risk groups with all genes comparison information. The significant enriched GO terms in the low-risk group were GOBP ALPHA BETA T CELL ACTIVATION, GOBP ANTIGEN RECEPTOR MEDIATED SIGNALING PATHWAY, GOBP IMMUNE RESPONSE REGULATING CELL SURFACE RECEPTOR SIGNALING PATHWAY, GOBP IMMUNE RESPONSE REGULATING SIGNALING PATHWAY, GOBP T CELL RECEPTOR SIGNALING PATHWAY, GOCC T CELL RECEPTOR COMPLEX, et al. (**Figure 8B**). The significant enriched HALLMARK terms in the low-risk group were HALLMARK ALLOGRAFT REJECTION, HALLMARK IL2 STAT5 SIGNALING, HALLMARK IL6 JAK STAT3 SIGNALING, HALLMARK INTERFERON ALPHA RESPONSE, HALLMARK INTERFERON GAMMA RESPONSE, HALLMARK PROTEIN SECRETION, et al. (**Figure 8C**).

### The relationship between riskscore and tumor microenvironment

**Figure 9:**
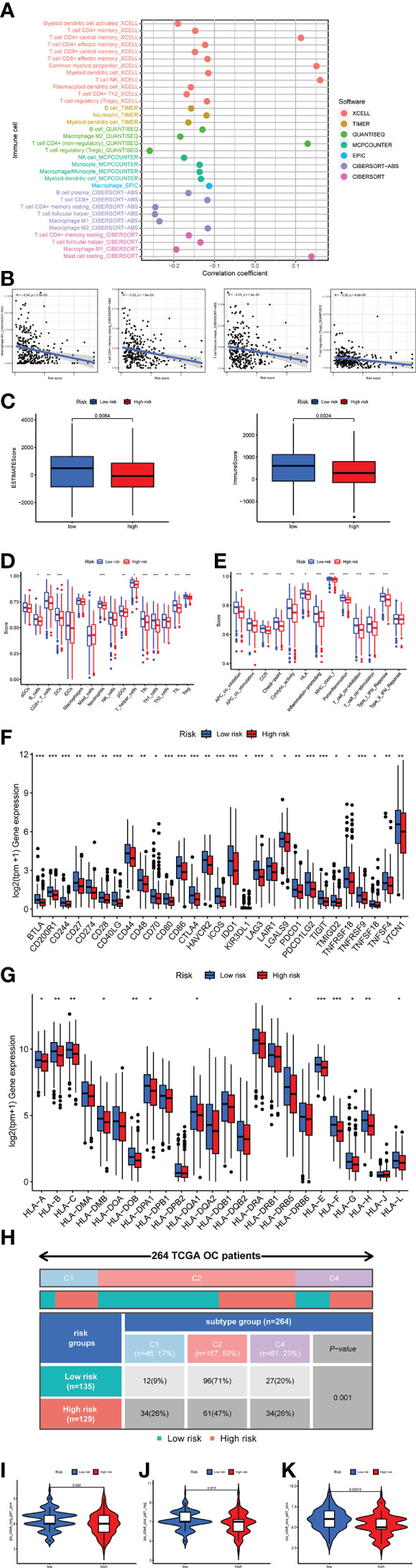
Investigation of tumor microenvironment in the high-risk and low-risk subgroups. **(A)** Correlation bubble plot of the abundance of the immune cells infiltration levels with riskscore. **(B)** A negative association between immune infiltration and risk score. **(C)** Comparison of immune-related scores between the low- and high-risk groups. **(D, E)** Enrichment analysis of immune cell infiltration and immune-related pathways. **(F)** The difference in the checkpoint expression between the risk groups. **(G)** The difference in the checkpoint expression between the risk groups. **(G)** The difference in the expression of human leukocyte antigen (HLA) genes between the risk groups. **(H)** Immune subtype difference between the risk groups. **(I)** Immunophenoscore (IPS) for immunotherapy. **(I)** CTLA4− PD1+. **(J)** CTLA4+ PD1−. **(K)** CTLA4+ PD1+.

It was essential to exploit the role of TME in ovarian cancer progression and metastasis to discover novel therapeutics for this deadly disease due to the successful drugs targeting TME. **Figure 9A** showed the correlation between immune infiltration level and riskscore based on the TIMER, CIBERSORT, CIBERSORT_ABS, QUANTISEQ, MCPCOUNTER, XCELL, and EPIC algorithms. It was easy to find that most immune cell infiltration levels were negatively correlated with riskscore (**Figure 9B**, **Supplementary Table 8**). Such as Macrophage M1, T cell CD4+ memory resting, and T cell follicular helper by algorithm CIBERSORT−ABS, T cell regulatory (Tregs) by algorithm QUANTISEQ (**Figure 9B**). We assessed immune scores and estimate scores in OC based on the estimate algorithm, and we found that low-risk groups tended to have higher scores (**Figure 9C**). Additionally, we used the ssGSEA to examine the distribution of immune cell infiltration and the enrichment of immune-related functional pathways in high-risk and low-risk subgroups, it was obvious that the majority of immune cell infiltration levels were significantly higher in the low-risk group and immune-related functional pathways were significantly enriched in the low-risk group (**Figures 9D, E**). We also found almost all immune checkpoints exhibited higher expression in the low-risk group, such as CD274, CD28, and LAG3 (**Figure 9F**). Human leukocyte antigen (HLA) genes were essential in antigen presentation. Our results also implied that most HLA genes had high expression levels in the low-risk group (**Figure 9G**). Thorsson et al. dentified six immune subtypes in 33 diverse cancer types, which was a resource for exploring immunogenicity in cancer. There was a significant immune subtypes composition difference between high-risk and low-risk groups (**Figure 9H**), indicating the different TME among the two risk groups. The above results proved that the riskscore was closely related to TME and time in OC patients. Therefore, we further explored the role of riskscore in immunotherapy through the TCIA database. The results indicated that the patients in the low-risk group were more sensitive to immunotherapy (**Figures 9I–K**).

### Mutation and chemotherapeutic drug responses

We assessed the top fifteen mutated genes in both risk groups. The oncoplot presented that most genes had different mutation frequency in the low-risk than high-risk group, such as genes APOB, FLG2 had higher mutation frequency in the low-risk (**Supplementary Figures 9A, B**). We also evaluated chemotherapeutic drug responses in patients of two groups. The results showed that chemotherapeutic drugs had lower half-maximal inhibitory concentration (IC50) in the low-risk group, such as ML323, Pictilisib, and Ruxolitinib (**Supplementary Figures 9C**).

## Discussion

In the world, OC is the leading cause of mortality among gynecologic malignancies with a high mortality on incidence ratio, accounting for the greatest proportion of gynecologic cancers. Although after primary treatment with surgery resection and chemotherapy, most patients achieved a complete response, 65-80% succumbed to recurrence with chemotherapeutic resistance in the first five years. In the past two decades, growing evidence suggested that immunotherapies have been widely used in the clinical treatment of various tumors. Despite treatments in cancer vaccines (such as BVX-0918), immune modulators (such as checkpoint inhibitors and cytokines), targeted antibodies (such as monoclonal antibodies), adoptive cell therapy (such as chimeric antigen receptor (CAR)- and TCR-engineered T cells) have been rapidly developing, immunotherapy response rates among ovarian cancer patients remained modest. Therefore, there was still a need to explore other biomarkers that may facilitate the not responded patients. The combination of therapeutic immunotherapy and chemotherapeutic therapy may improve treatment efficiency significantly.

Cytotoxic T cells were essential effectors of anti-tumor immunity. CD4+ T cell refered to a population of T lymphocytes which exhibited T cell receptors (TCRs) that specifically recognized peptide antigens presented in association with Class II major histocompatibility complex (MHC II) molecules. CD4+ T cell were remarkably versatile and possessed multifunctional characteristics. These cells made up the secondary component of adaptive T cell-mediated immunity. In response to signals that varied based on the situation, CD4+ T cells had the ability to differentiate into multiple distinct functional subtypes. In response to signals that vary based on the situation, CD4+ T cells have the ability to differentiate into multiple distinct functional subtypes (51, 52). Much of the previous studies have put the focus of research on CD8 T cell instead of CD4 T cell function in cancer (53–55). Most insights into CD4+ T cells have focused on anti-viral immunity and autoimmunity, such as human cytomegalovirus (56, 57), epstein-barr virus (58), and autoimmune encephalomyelitis (59). In recent years, multiple studies have demonstrated that CD4+ T cells are critical to the response to cancer immunotherapy. Kwek et al. revealed pre-existing levels of PD-1+CD4+ T cells instead of CD8 + T cells in the circulation associated with improved overall survival in prostate cancer patients treated with ipilimumab (15). Cohen first discovered that B cell maturation antigen-specific chimeric antigen receptor (CAR) T cells reponse were positively associated with higher premanufacturing CD4/CD8 T cell ratio in multiple myeloma (18). The neoantigen vaccination derived from RNA-seq and whole-exome sequencing datasets that were currently of interest to major pharmaceutical companies, the neoantigens recognized by CD4 T cell and MHC class II-restricted manner played a vital role in the recovery of cancer patients (60, 61).

Currently, there have been many predictive signatures developed to predict patient prognosis outcomes for a better understanding of precision genomic medicine. Such as immune-related genes risk signature in glioblastomas (62), cuproptosis-related genes risk signature in hepatocellular carcinoma (63), ferroptosis-related genes signature in hepatocellular carcinoma (64). However, there were a handful of known studies with CD4 T cells related signatures, such as CD4+ conventional T cells-related lncRNA signature in breast cancer and hepatocellular carcinoma prognosis (23, 24). Recent applications of scRNA-seq in dissecting TME have allowed a detailed understanding of the biology of tumor-infiltrating immune cells properties of heterogeneity and potential roles in both tumor progression and response to immune checkpoint inhibitors and other immunotherapies. In the present study, we constructed a novel risk signature to predict prognosis and survival for OC based on the CD4+ conventional T cells-related genes based on scRNA-seq and TCGA bulk-seq datasets. Internal validation was conducted firstly by splitting the TCGA bulk-seq datasets into train and test at a ratio of 1:1. We then validated the risk signature OS and PFS in another three GEO datasets. This result proved that our risk signature was robust. The risk signature was an independent prognostic factor through multivariate Cox regression analysis. Nomogram was used to improve the clinical unity of riskscore. Calibration curve, DCA, and ROC were performed to test the accuracy of the risk signature. Furthermore, we compared our model with some models reported in the past and found our model was better in 1 year,found our model was better in 1 year, 3 years and 5 years. We also found that there were significant differences in the expression of many immune checkpoint genes expression, some of which promoted immunity and some inhibited immunity. Among them, the survival condition of patients in the high-risk group was even worse, which may be due to the formation of an immunosuppressive microenvironment in this group of patients.We also expanded the risk signature to immunotherapy by thoroughly analysing the TME status difference between high-risk and low-risk groups. Chemotherapeutic drugs were also examined among high-risk and low-risk groups.

However, this study had certain limitations. Firstly, the present findings require further prospective validation by multicenter study cohorts. Secondly, further study of the functions and molecular mechanisms of these 11 CD4TGs in combination with more *in vitro* and *in vivo* experiments were required in OC. Nonetheless, we provided clues to identify CD4TGs that could be used as potential prognostic biomarkers and therapeutic targets with a good clinical prediction value.

## Conclusion

Overall, we identified 11 CD4TGs involved in a risk model as a biomarker in OC based on scRNA-seq datasets, TGCA bulk-seq datasets and GEO probe datasets. Significant differences in survival rate and TME status were observed between the high-risk and low-risk groups, thus implying useful information for predicting clinical outcomes and may become a therapeutic target for patients with OC. As the nature of cancer immunotherapy was increasingly revealed, our study may provide new ideas on the role of CD4TGs in treating OC.

## Data availability statement

The datasets presented in this study can be found in online repositories. The names of the repository/repositories and accession number(s) can be found within the article/**Supplementary Material**.

## Author contributions

TH, D-XL, X-CZ, S-TL, and PY performed data collection and analysis. The study design and manuscript edition was done by TH, QZ, and S-BC, with all authors contributing to previous versions of the manuscript. All authors contributed to the article and approved the submitted version.

## Supplementary material

Supplementary Figure 1 The expression of classical markers across different subsets in two single-cell RNA-seq datasets. **(A)** The expression of some classical markers on dotplot, **(B)** umap, **(C)** violin plots across different cell subsets in dataset GSE118828. **(D)** The expression of some classical markers on dotplot, **(E)** umap, **(F)** violin plots across different cell subsets in dataset GSE147082.

Supplementary Figure 2 Functionally enriched KEGG pathways. **(A)** The heatmap showed functionally enriched up-regulated KEGG pathways identified based on differential genes in each cell type in dataset GSE118828. **(B)** The heatmap showed functionally enriched down-regulated KEGG pathways identified based on differential genes in each cell type in dataset GSE118828. **(C)** The heatmap showed functionally enriched up-regulated KEGG pathways identified based on differential genes in each cell type in dataset GSE147082. **(D)** The heatmap showed functionally enriched down-regulated KEGG pathways identified based on differential genes in each cell type in dataset GSE147082.

Supplementary Figure 3 The single-cell metabolic features of cell subsets. **(A)** The metabolic status of different clusters of cell types in dataset GSE118828. **(B)**. The single-cell metabolic features of cell subsets in dataset GSE147082.

Supplementary Figure 4 The ROC of other previous established risk models at 1 year, 3 years and 5 years. **(A)** 3 lncRNA risk model. **(B)** 5 lncRNA risk model. **(C)** 6 lncRNA risk model. **(D)** 8 mRNA risk model. **(E)** 12 mRNA risk model.

Supplementary Figure 5 Analyzing and Estimating Nomogram. **(A)** Nomogram that integrated the riskscore, age, grade, stage, and tumor residual size predicted the probability of the 1-, 3-, and 5-year OS. **(B)** Calibration curves analysis for 1-, 3-, and 5-year OS. **(C)** decision curve analysis (DCA) of the nomogram in TCGA whole dataset for evaluating the clinical usefulness in 1-year OS.

Supplementary Figure 6 The expression levels of the genes selected for risk pattern analysis in single-cell dataset. **(A)** In single-cell dataset GSE118828 by dotplot. **(B)** In single-cell dataset GSE118828 by violin plot. **(C)** In single-cell dataset GSE147082 by dotplot. **(D)** In single-cell dataset GSE147082 by violin plot. **(E)** The risk score calculated by AddModuleScore function was displayed in cell subsets level in datasets GSE118828 and GSE147082. **(F)** The risk score calculated by AddModuleScore function was displayed in sample level in datasets GSE118828 and GSE147082.

Supplementary Figure 7 The risk genes expression in bulk-seq datasets. **(A)** In dataset TCGA by violin plot. **(B)** In dataset GSE63885 by violin plot. **(C)** In dataset GSE26193 by violin plot. **(D)** In dataset GSE147082 by violin plot.

Supplementary Figure 8 The risk gene expression in two ovarian cancer celllines (SKOV3, A2780) and one normal ovarian celline (ISOE). **(A)** The 11 risk genes expression, and were normalized to gene expression in celline ISOE.

Supplementary Figure 9 Mutation and Chemotherapeutic Drug Responses. **(A, B)** Top fifteen mutated genes frequency in both risk groups. **(C)** Chemotherapeutic drug half-maximal inhibitory concentration (IC50) in patients of two groups.

Supplementary Table 1 Cell markers of cell subsets.

Supplementary Table 2 The list of CD4+ conventional T cells-related genes.

Supplementary Table 3 The genes list after performing univariate Cox regression analysis.

Supplementary Table 4 Clinical features comparison between high-risk and low-risk subgroups.

Supplementary Table 5 The concrete clinical information for TCGA whole dataset patients.

Supplementary Table 6 The detailed risk genes expression, riskscore and risk group in all models.

Supplementary Table 7 Differential genes information between high-risk and low-risk subgroups.

Supplementary Table 8 Profile of significantly different infiltrated immune cells between high-risk and low-risk subgroups by different algorithms.

Supplementary Table 9 11 risk genes primer sequences.
