# Supplementary figures and images for: CD4+ conventional T cells-related genes signature is a prognostic indicator for ovarian cancer
Source: Front Immunol. 2023 Mar 31;14:1151109. doi: 10.3389/fimmu.2023.1151109 (PMC10104164; doi:10.3389/fimmu.2023.1151109)

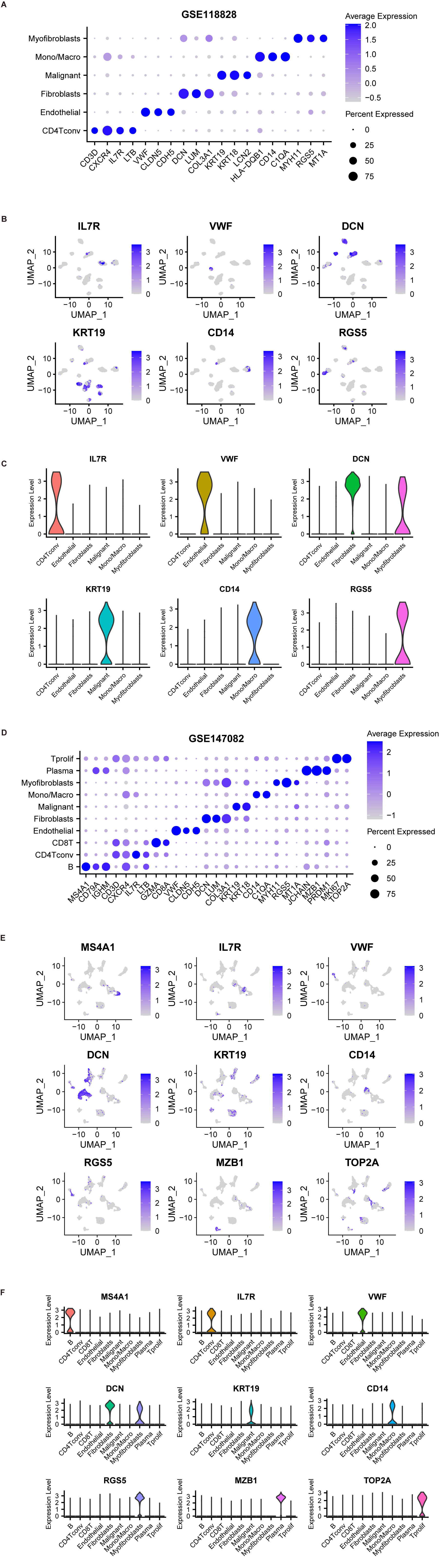

Supplement: Supplementary Figure 1 — The expression of classical markers across different subsets in two single-cell RNA-seq datasets. (A) The expression of some classical markers on dotplot, (B) umap, (C) violin plots across different cell subsets in dataset GSE118828. (D) The expression of some classical markers on dotplot, (E) umap, (F) violin plots across different cell subsets in dataset GSE147082. [file Image_1.tif]

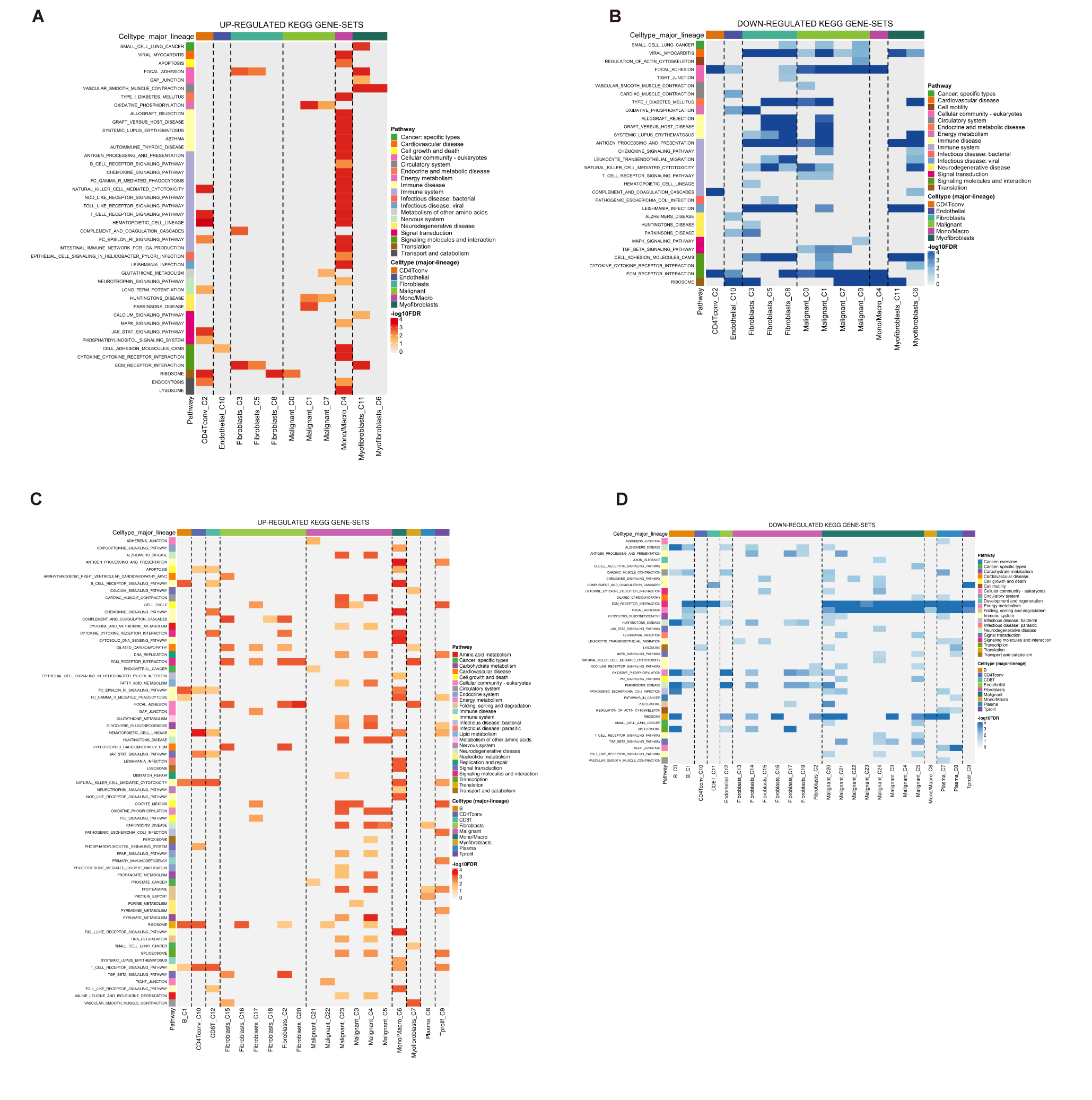

Supplement: Supplementary Figure 2 — Functionally enriched KEGG pathways. (A) The heatmap showed functionally enriched up-regulated KEGG pathways identified based on differential genes in each cell type in dataset GSE118828. (B) The heatmap showed functionally enriched down-regulated KEGG pathways identified based on differential genes in each cell type in dataset GSE118828. (C) The heatmap showed functionally enriched up-regulated KEGG pathways identified based on differential genes in each cell type in dataset GSE147082. (D) The heatmap showed functionally enriched down-regulated KEGG pathways identified based on differential genes in each cell type in dataset GSE147082. [file Image_2.tif]

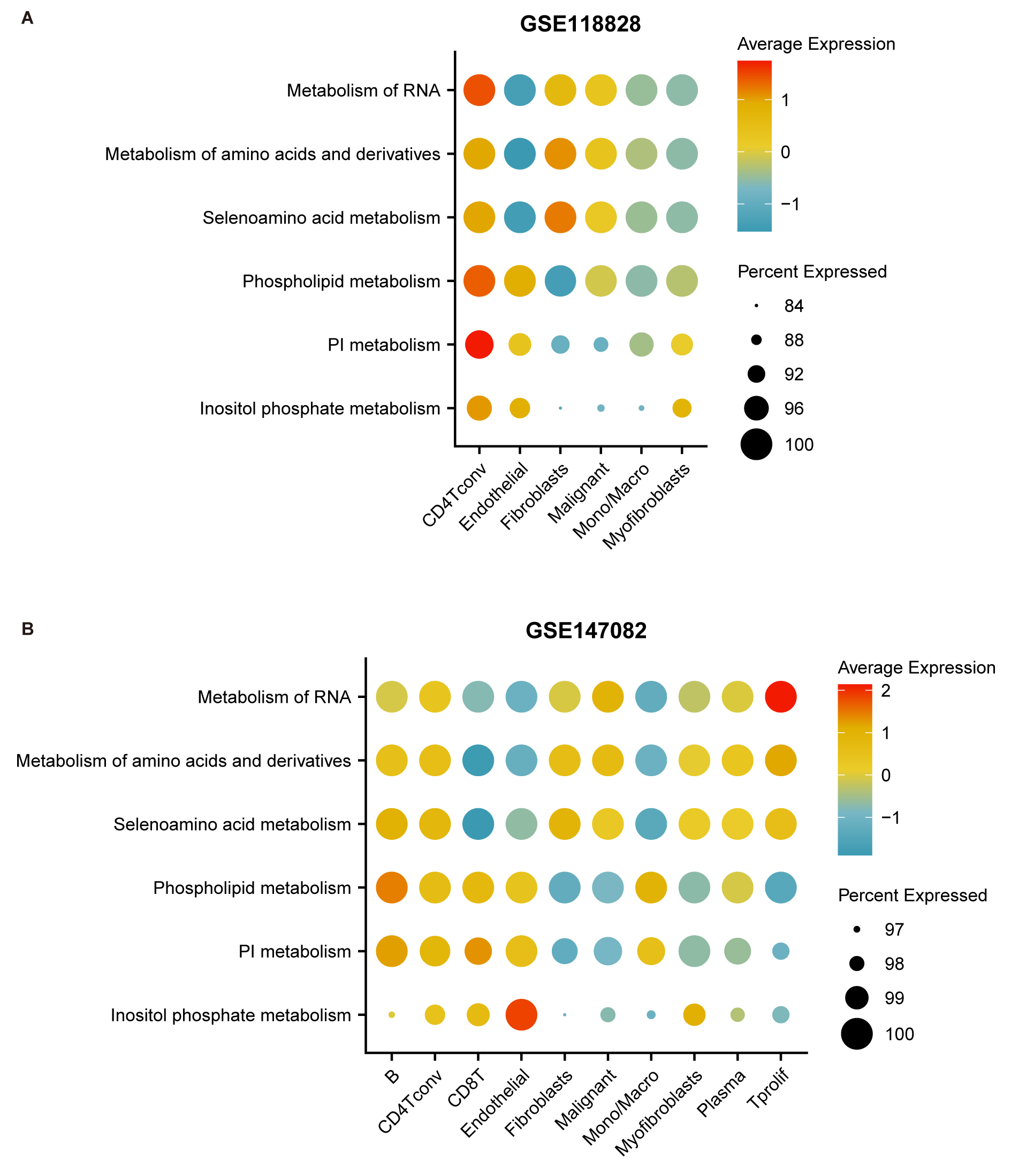

Supplement: Supplementary Figure 3 — The single-cell metabolic features of cell subsets. (A) The metabolic status of different clusters of cell types in dataset GSE118828. (B). The single-cell metabolic features of cell subsets in dataset GSE147082. [file Image_3.tif]

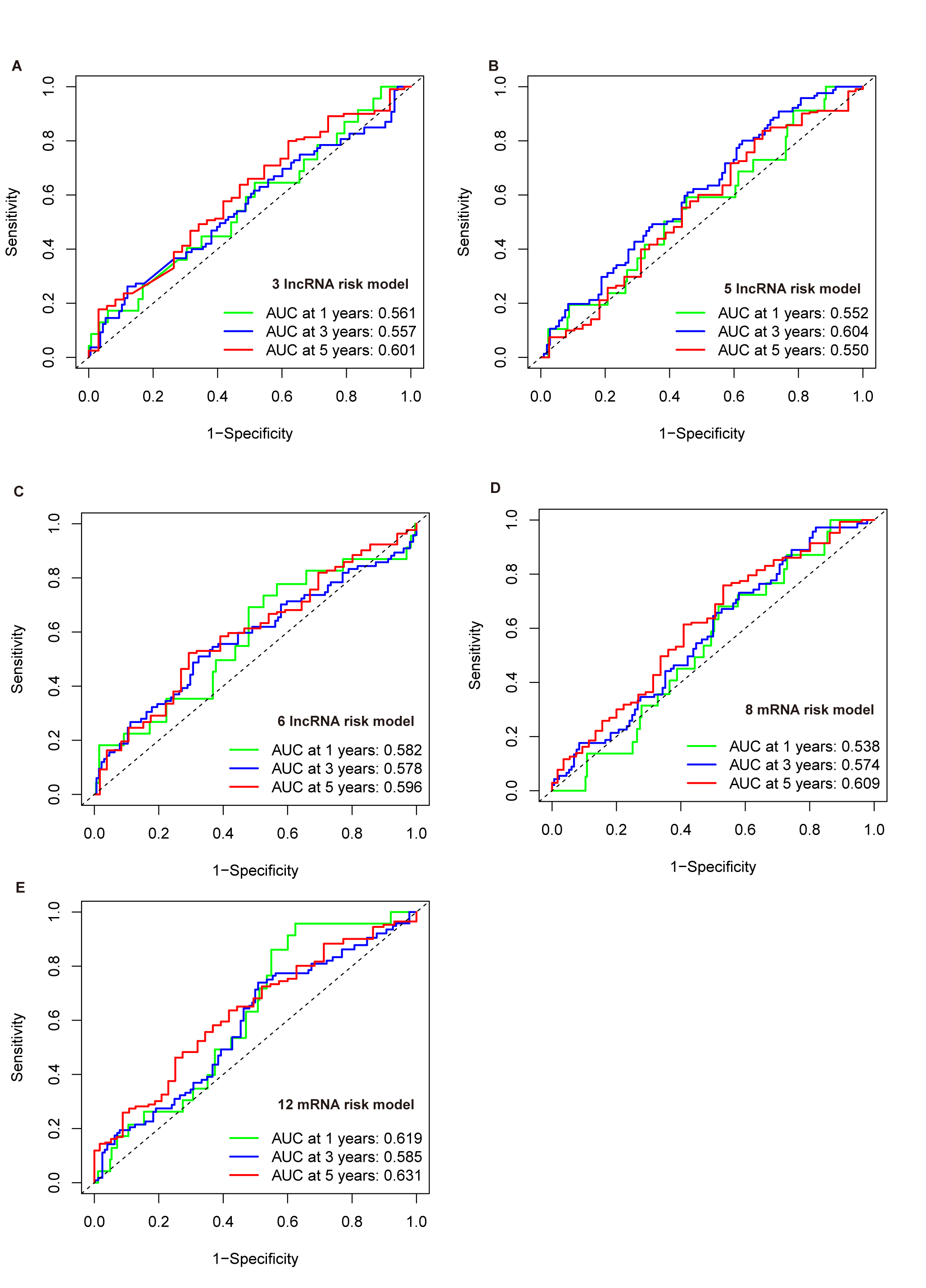

Supplement: Supplementary Figure 4 — The ROC of other previous established risk models at 1 year, 3 years and 5 years. (A) 3 lncRNA risk model. (B) 5 lncRNA risk model. (C) 6 lncRNA risk model. (D) 8 mRNA risk model. (E) 12 mRNA risk model. [file Image_4.tif]

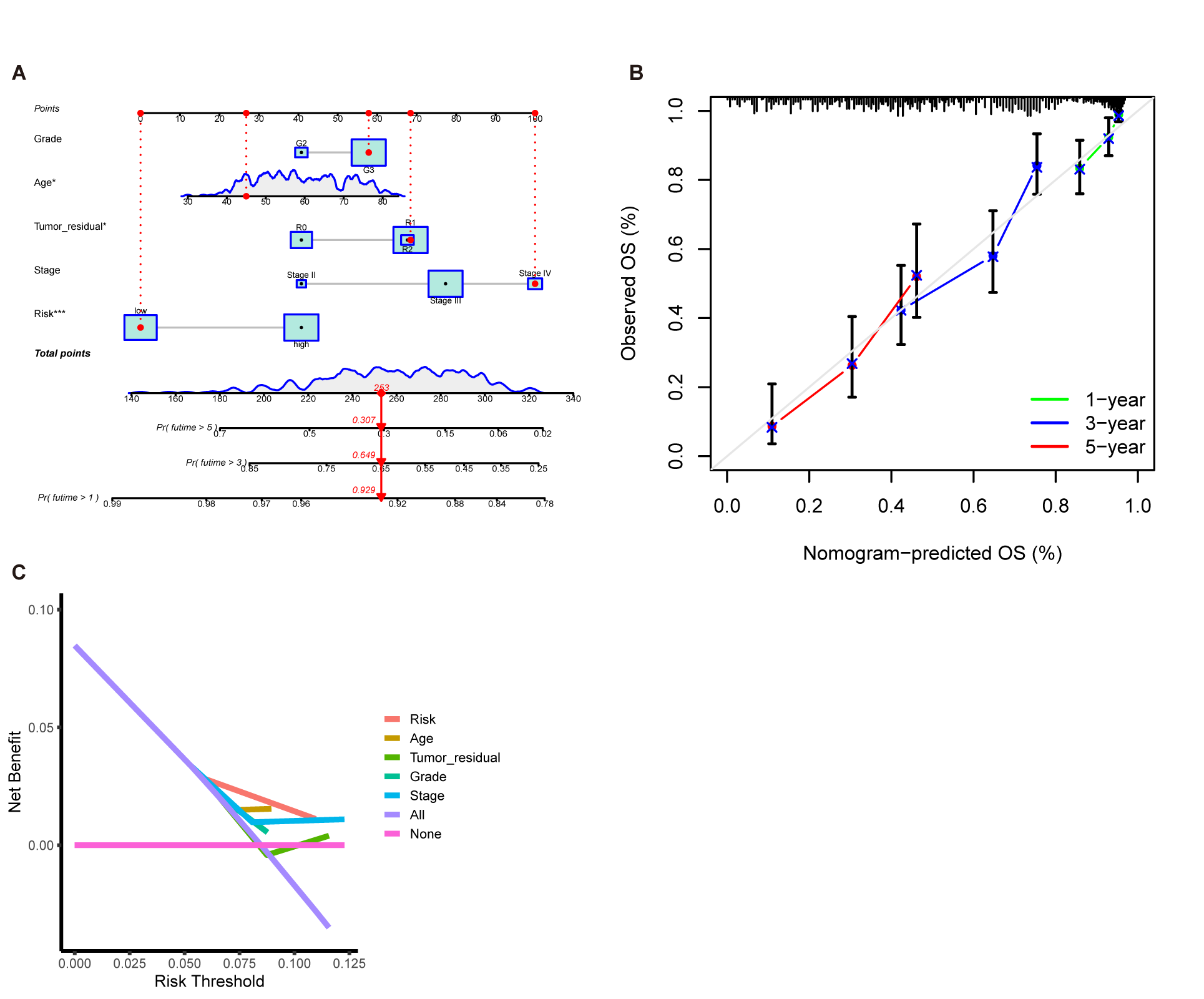

Supplement: Supplementary Figure 5 — Analyzing and Estimating Nomogram. (A) Nomogram that integrated the riskscore, age, grade, stage, and tumor residual size predicted the probability of the 1-, 3-, and 5-year OS. (B) Calibration curves analysis for 1-, 3-, and 5-year OS. (C) decision curve analysis (DCA) of the nomogram in TCGA whole dataset for evaluating the clinical usefulness in 1-year OS. [file Image_5.tif]

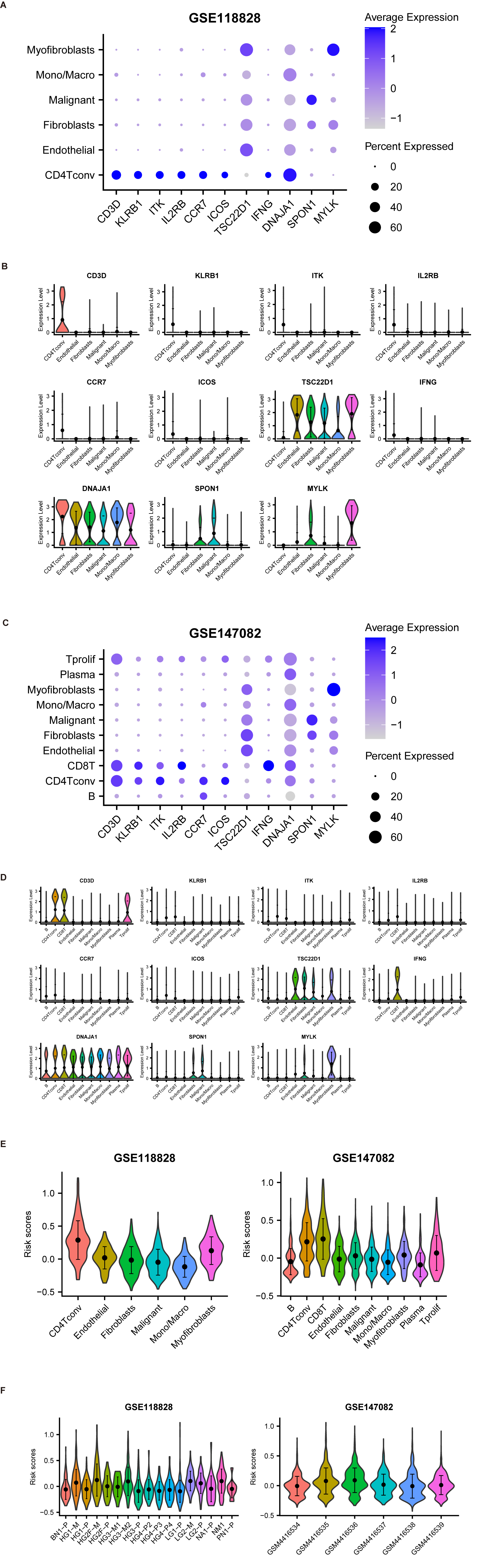

Supplement: Supplementary Figure 6 — The expression levels of the genes selected for risk pattern analysis in single-cell dataset. (A) In single-cell dataset GSE118828 by dotplot. (B) In single-cell dataset GSE118828 by violin plot. (C) In single-cell dataset GSE147082 by dotplot. (D) In single-cell dataset GSE147082 by violin plot. (E) The risk score calculated by AddModuleScore function was displayed in cell subsets level in datasets GSE118828 and GSE147082. (F) The risk score calculated by AddModuleScore function was displayed in sample level in datasets GSE118828 and GSE147082. [file Image_6.tif]

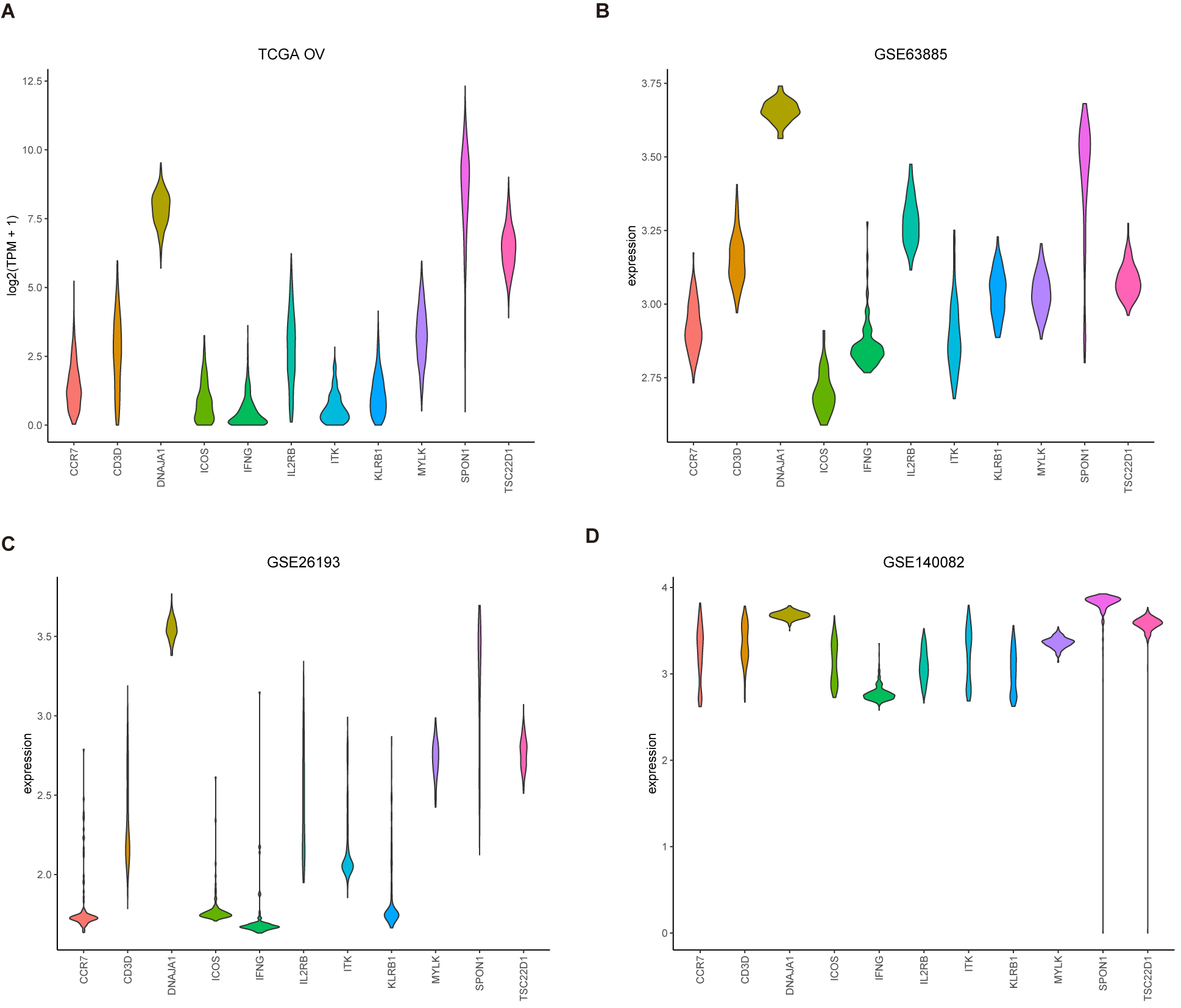

Supplement: Supplementary Figure 7 — The risk genes expression in bulk-seq datasets. (A) In dataset TCGA by violin plot. (B) In dataset GSE63885 by violin plot. (C) In dataset GSE26193 by violin plot. (D) In dataset GSE147082 by violin plot. [file Image_7.tif]

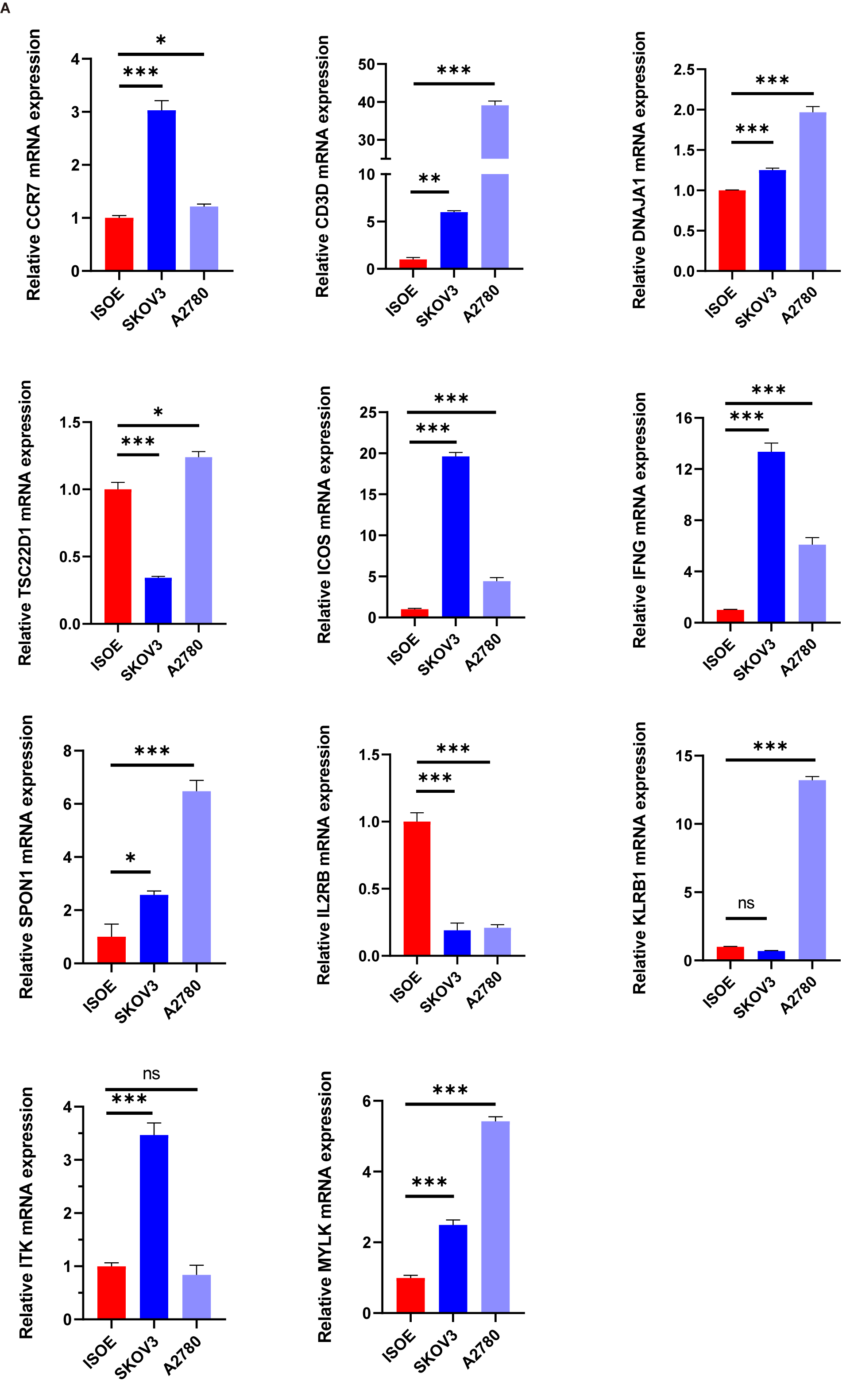

Supplement: Supplementary Figure 8 — The risk gene expression in two ovarian cancer celllines (SKOV3, A2780) and one normal ovarian celline (ISOE). (A) The 11 risk genes expression, and were normalized to gene expression in celline ISOE. [file Image_8.tif]

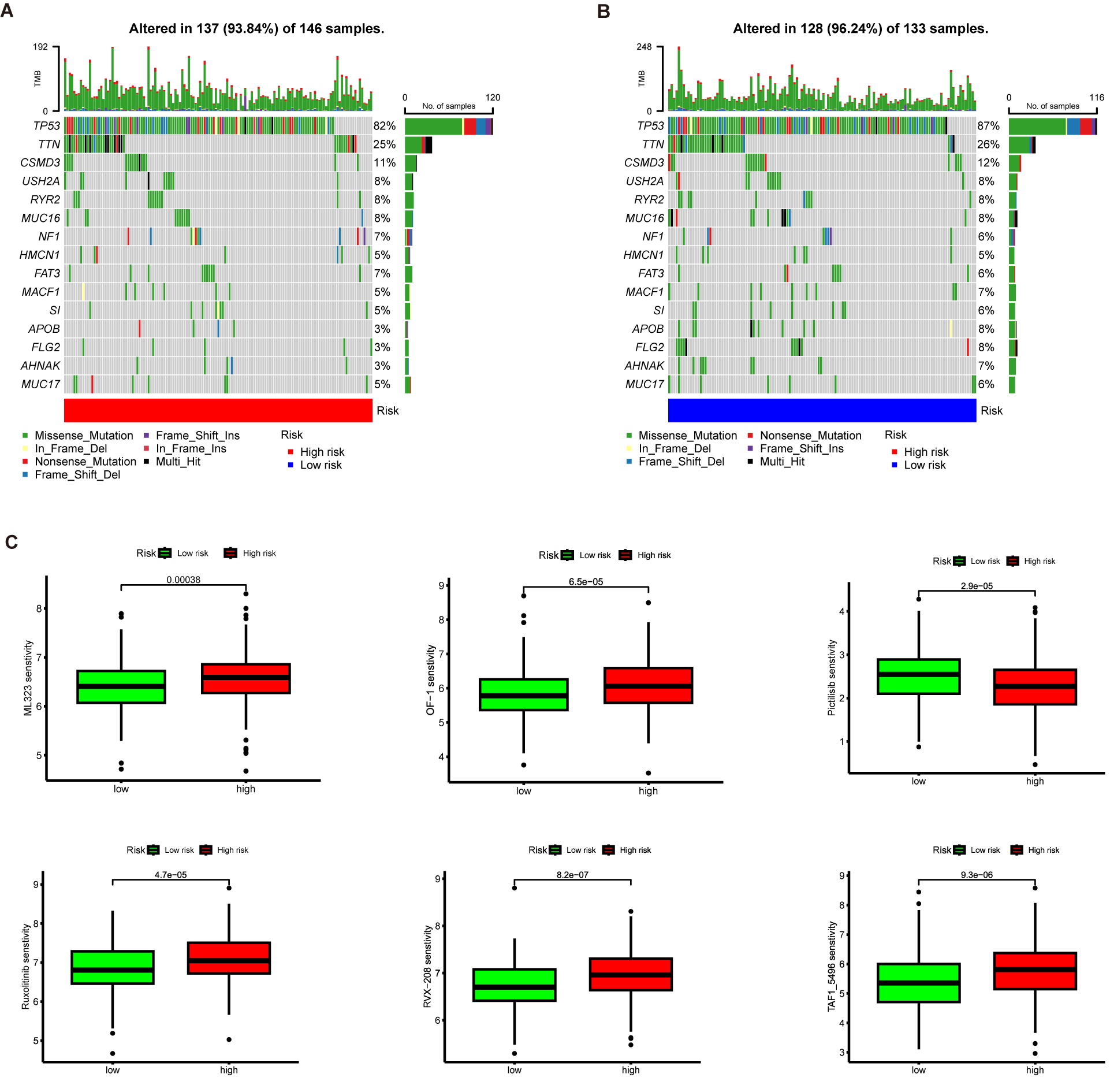

Supplement: Supplementary Figure 9 — Mutation and Chemotherapeutic Drug Responses. (A, B) Top fifteen mutated genes frequency in both risk groups. (C) Chemotherapeutic drug half-maximal inhibitory concentration (IC50) in patients of two groups. [file Image_9.tif]
